# Research on target detection based on improved YOLOv7 in complex traffic scenarios

**DOI:** 10.1371/journal.pone.0323410

**Published:** 2025-05-19

**Authors:** Yuhang Liu, Huibo Zhou, Ming Zhao

**Affiliations:** School of Mathematical Sciences, Harbin Normal University, Harbin, Heilongjiang Province, 150500, China; Sichuan University, CHINA

## Abstract

Target detection is an essential direction in artificial intelligence development, and it is a crucial step in realizing environmental awareness for intelligent vehicles and advanced driver assistance systems. However, the current target detection algorithms applied to complex real-life scenarios still have a lot of intractable problems, such as the detection of different road scenarios, not having a good real-time detection capability, and so on. Therefore, there is a need to balance the efficiency and effectiveness of the target detector. YOLOv7, as a single-stage target detection algorithm, combines a number of advanced modules and methods for the purpose of achieving more precise and faster target detection. This paper, YOLOv7 is used as a baseline, combined with deformable convolution, to realize adequate detection in complex scenes and balanced computational efficiency and accuracy by adding an attention mechanism module. In addition, combining the lightweight network module speeds up the model’s computational speed while improving the detector’s feature expression ability, thus accomplishing the task of real-time target detection in complex traffic scenes. Compared with YOLOv7, our model improves the average accuracy by 3.7% on the SODA 10M dataset, and the mean average precision (mAP) value reaches 75.9%.

## Introduction

This paper investigates the problem of recognizing intelligent traffic targets in complex scenes that have emerged in computer vision in recent years. Intelligent information processing research has always considered urban traffic safety as an important research object. With the implementation of the Made in China 2025 strategy, the field of artificial intelligence in computer science has been developing rapidly, and computer vision is a critical part of the field of artificial intelligence. It is an indispensable technology in the construction of intelligent transportation as well as smart cities [1].

In recent years, target detection technology, as a hot topic and focus in the direction of machine vision, has experienced the process of development from machine learning to deep learning, from just starting to the wide application [2], which aims to allow computers to automatically extract the specified features of the detected target through training and see special features, and has essential application significance in numerous aspects such as automatic driving, medical monitoring, and human-computer interaction. In the research area of target detection, road detection technology has been emphasized by many research institutes, colleges, and universities at home and abroad and has become the object of exploration by many researchers in the academic world.

For complex traffic scenarios, the target detection algorithm needs to satisfy two conditions. First, it needs to have high precision to detect road targets; second, an instantaneous detection rate is crucial for intelligent transportation systems. Currently, the application of target detection technology in complex traffic scenarios is mainly divided into two mainstream directions: one is the traditional detection algorithm based on machine learning, and the other is the deep learning detection algorithm based on convolutional neural networks [3,4]. Standard target detection algorithms are usually divided into three stages: regional options, feature extraction, and characteristic classification [5]. The purpose of area selection is to determine the location of the target, which may vary in position and aspect ratio in the image. This phase usually involves a sliding window strategy to iterate over the entire image [6], taking into account different aspect ratios and proportions. Next, feature extraction algorithms such as Histogram of Oriented Gradients (HOG) [7] and Scale Invariant Feature Transform (SIFT) [8] will be used to extract the corresponding features. Eventually, classifiers such as Support Vector Machine (SVM) [9] and Adaptive Boost (Ada-Boost) [10] are used to categorize the extracted features. However, the target detection algorithms based on traditional manual parts and machine learning have issues such as too much computation, inaccuracy, difficulty to design, poor robustness, etc., in actual vehicle detection, which cannot meet the detection needs in complex traffic environments.

Convolutional neural network-based target detection algorithms show better performance in both feature extraction and modeling for the target detection task, and in a large number of related studies, traditional target detection algorithms have certain limitations in practical applications, while deep learning-based target detection algorithms have higher performance and accuracy and thus are more advantageous in practical scenarios [11]. The importance of deep learning-based target detection and semantic partitioning algorithms for target detection becomes prominent in complex road scenes [12]. Currently, deep learning-based target detection algorithms can be categorized into two groups. The first class is a two-stage detection algorithm based on candidate regions, representative algorithms include R-FCN (Region-based Fully Convolutional Networks) [13] and R-CNN (Region-CNN) family of algorithms (R-CNN [14], Fast-RCNN [15], Faster-RCNN [16], Mask-RCNN [17], Cascade-RCNN [18], etc.). The two-stage detectors have higher target detection accuracy but slower inspection speeds. The other category is single-stage detectors such as the YOLO (You Only Look Once) family of algorithms(YOLO [19], YOLO9000 [20], YOLOv3 [21], YOLOv4 [22], YOLOv5 [23], YOLOv6 [24], YOLOv7 [25]) and SSD (Single Shot MultiBox Detector) [26]. Classification of objects and bounding box regression can be performed at the same time without generating a region proposal, which directly causes the position coordinates of the class objects. Therefore, the single-stage detection algorithm meets the real-time requirements for detection speeds but suffers from lower detection accuracy than the two-stage detector [27]. For the detection of complex traffic scenes, it is essential to maintain a balance between computational precision and real-time inspection speed.

Although the target detection algorithms are on a path of continuous development, there are still many complex problems to be solved when they are applied to complex real-life scenarios, such as the issue of small target leakage, the problem of detecting targets in dense environments, the concern of occlusion, the problem of multi-scale target detection, etc., all of which need to be investigated in depth.

YOLOv7, as a rapid and efficient single-stage algorithm, demonstrates excellent potential for detecting targets in complex traffic scenes. However, the algorithm cannot be directly used to achieve satisfactory results by accurately reckoning the location and direction information of road objects. However, YOLOv7 redesigns the SPP module concerning the CSP structure for extracting features at various scales, leading to enhancing the robustness of the model. Meanwhile, to improve the efficiency of model training, YOLOv7 increases the number of positive samples. During the training process, each ground truth box can be predicted by multiple prior boxes, which enables the training to get the best shape-matching results. In terms of architecture, the ELAN-based Extended Efficient Layer Aggregation Network (E-ELAN) [28] is proposed, which is expanded, disorganized, and collocated to enhance e-learning abilities without compromising the initial gradient direction pathway.

In this article, we present a method based on an improved YOLOv7, named YOLOv7_ned, for target detection methods in complex traffic scenarios. We use the YOLOv7 algorithm as a baseline to achieve adequate target detection in complex scenes by backbone modeling with deformable convolutional DCN-v2 [29] and redesigning the YOLOv7 detection module. Meanwhile, the lightweight network module EVCBlock [30] is combined to balance computational efficiency and accuracy to achieve the task of real-time target detection in complex traffic scenarios. We performed extensive experiments on the unmanned dataset of SODA 10M [31], and the results show that our proposed improved YOLOv7 method is an efficient road target detection method.

Our unique contributions to this project are set out below:

1) Taking YOLOv7 as the benchmark, the deformable convolutional DCN-v2 is used on this foundation, which makes the modified algorithm well-adapted to a variety of complex traffic scenarios.
2) In order to improve road target recognition accuracy, the Normalized Attention Mechanism Module (NAM Attention) [32] is introduced to help the model make better use of the global contextual information for selecting essential regions in the image.
3) To increase the practicality of the improved algorithm and reduce the computational volume of the model, we employ EVCBlock, a lightweight network module, to enhance the feature representation of the model.

The remainder of this article is composed as follows: Part II describes the related work; Part III describes the primary methodology of the article; experiments are conducted; and results are given in Part IV. Part V discusses and summarizes the entire text.

## Related work

In this context, we first provide an introduction to data enhancement methods and select appropriate data enhancement methods for detection in complex traffic scenarios. At the same time, we have investigated the impact of the attention mechanism module on target detection.

### Data enhancement

The quality of dataset images greatly affects the ease of the subsequent feature extraction steps and determines the final detection of the framework. Data augmentation, also called data expansion, refers to the practice of obtaining equivalent value from a limited amount of data without substantially increasing the actual amount of data. Supervised data augmentation and unsupervised data augmentation are two types of these. Supervised data enhancement includes two further forms of single-sample and multi-sample data augmentation. Commonly used single-sample data enhancement methods are geometric transformations [33] (image translation transformations, image scale transformations, image rotation transformations, image folding transformations, etc.) and color transformations [34] (color channel transformations, adding noise, etc.). Geometric transformations of images, also known as image space transformations, map position coordinates in a single image to new position coordinates in the transformed image and are applied to matrix operations in mathematics. The color transformation approach is commonly used for training datasets with high color requirements, such as traffic light dataset detection in traffic systems, where amplifying the dataset through color channel transformation results in more accurate detection results. Different from single-sample data enhancement, multi-sample data enhancement utilizes multiple samples to produce new ones. This includes methods like Mosaic, SMOTE (Synthetic Minority Over-sampling Technique), and SamplePairing. Therefore, we adopted the mosaic data augmentation approach to augment the dataset to improve its quality. YOLOv7 greatly enriches the detection dataset by randomly adding and chopping four frames and assembling them into new pieces of training data. Factors such as mutual occlusion between vehicles and pedestrians or the excessive distance of the photographed target area make the target features significantly different under different conditions, which in turn affects the quality of the dataset samples. However, mosaic is able to dramatically improve the non-uniform distribution of objects in the SODA 10M dataset. The randomness of its splicing makes the network more robust. Furthermore, mosaic augmentation can compute the data of the four images in a direct way, which gives better performance using a single GPU and well solves the problem of high demand on GPU resources [35].

### Attention mechanism module

The attention mechanism is a standard data processing method that is extensively used in various machine learning task domains. In computer vision, the key idea at the heart of the attention mechanism is to discover correlations between raw data and emphasize essential characteristics such as multi-order attention, pixel attention, channel attention [36], etc. In the area of target detection in complex traffic scenarios, the real-time nature of detection plays a crucial role. Using the attention-based approach, one can avoid manual labeling of regions by selecting the significant regions in the image by utilizing the attention attribute of the CNN feature maps. For the past few years, models based on the self-attention mechanism have obtained comparable or even better results than CNNs on many visual tasks. Earlier studies, such as those shown by SENet [37] and CBAM [38], can be used as an enhancement of the convolution module. Recently, self-attention has been presented as a stand-alone module in place of the traditional convolutional CNN modules, e.g., SAN [39] and BoTNet [40]. These works aim to devise more flexibility in the design of feature extractors by pooling information from a more extensive set of particles.

## Methods

### Overview

**Fig 1:**
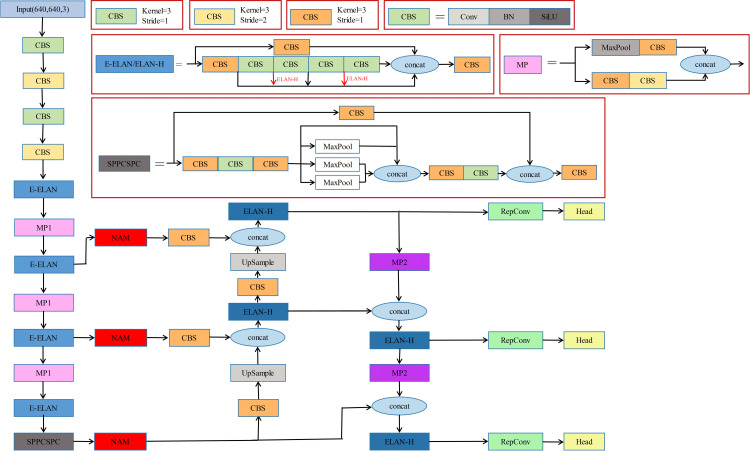
The network architecture of YOLOv7_ned.

An anchor-based single-stage detector usually consists of INPUT, BACKBONE, and HEAD for object classification and localization. As shown in (Fig 1), we outline our approach. In this article, we have changed the detailed architecture of YOLOv7 by replacing the final stage 3*3 convolutional layer using deformable convolution DCN-v2 to achieve accurate detection in complex traffic scenes. The attention module NAM Attention is added to the SPP module and FPN module to adapt the weight of the feature maps of different layers so that the network focuses more on the small target features, such as vehicles and pedestrians, to improve precision and robustness. Meanwhile, the lightweight module EVCBlock is used to minimize the network parameter count, computational complexity, and redundancy to balance the model’s computational accuracy and real-time performance.

### Select YOLOv7 as the baseline

A deep learning-based target detector usually consists of a data loader (image preprocessing), backbone (extracting target features), neck (collecting and combining target features), head (prediction part), and loss function. In the structure of YOLOv7, still based on the anchor-based approach, the REP layer is added to the network architecture along with the E-ELAN layer. The SPP module is redesigned concerning the CSP architecture to facilitate subsequent deployment, and it also contains a global average pooling layer for aggregating all features into a fixed-size vector. This approach significantly reduces the number of network participants while increasing the remaining feature information and enhancing feature learnability.

The backbone of YOLOv7 utilizes an experimental, innovative multi-branch stacking design for feature acquisition, which makes the model more densely populated with jump-connected structures compared to the previous Yolo. An experimental downsampling structure is also used, where features are extracted and compressed in parallel using Maxpooling and a step size of 2x2.

As with the backbone section, the enhanced feature extraction section also uses a multi-input stacking structure for feature extraction. The architecture of Panet is still adopted in YOLOv7, which not only up-sample the characteristics for feature integration but even down-samples the features for feature fusion again.

Meanwhile, YOLOv7 uses SPP with a CSP structure to enlarge the receptive field, and a CSP module is added to the SPP structure, which has a sizeable margin of residuals to help with optimization and feature acquisition. The number of positive samples has been increased in YOLOv7 to expedite the model training efficiency, and each accurate frame can be predicted by more than one a priori structure responsible for the prediction at the time of training. In addition to this, for each authentic frame, the IoU and kind of prediction frames adjusted to the a priori frames are also calculated to obtain the cost, which in turn finds the most suitable a priori frame for that true frame.

To reduce the network parameters, YOLOv7 also borrows the structure of RepVGG and introduces RepConv in specific parts of the network. Therefore, YOLOv7 has excellent potential for improving the accuracy of complex object detection, and utilizing YOLOv7 as a baseline algorithm for improving object detection in complex traffic scenes is very beneficial.

### ECVBlock

**Fig 2:**
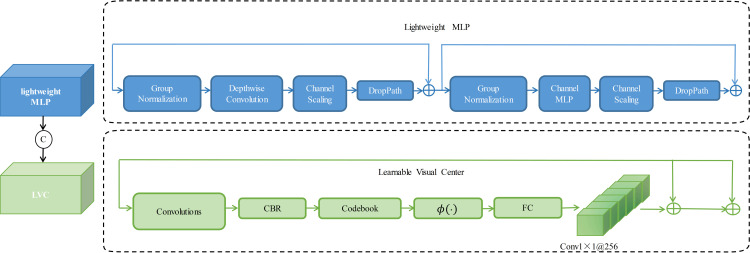
Schematic representation of the explicit visual center.


For application scenarios that demand high real-time processing capability, such as autonomous driving or automobile-assisted driving, it is essential to enhance the computational speed and precision of target detection further. Adding EVCBlock to CFPNet is an improved method for target detection tasks, which can increase the computational productivity and detection precision of the model. EVCBlock is a lightweight network module that speeds up the computation of the model while enhancing its feature representation. As shown in (Fig 2), EVCBlock mainly consists of two modules linked in tandem. Firstly, a lightweight MLP is employed to catch the global long-term dependencies of the top-level feature $X_{4}$. It is proposed to implement the LVC (learnable visual center) mechanism on $X_{4}$ to snap a localized angle area of the input image. Between $X_{4}$ and EVC, a Stem block is used to smooth the features instead of implementing it directly on the original feature map, as in YOLOv5. The above process can be expressed as follows:

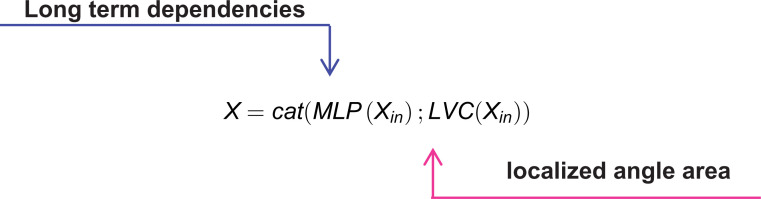


In this context, the output of EVC is denoted as X and Cat(·) represents the concatenation of feature maps along the dimension of the channel. $\;MLP\left(X_{in}\right)$ and $LVC(X_{in})$, respectively, denote the output characteristics of the lightweight MLP and LVC mechanisms. $\;X_{in}$ represents the output of the Stem program block, with the calculation formula given by:


$$X_{in}=\sigma (BN({Conv}_{7\times 7}(X_{4})))$$

In this case, ${Conv}_{7\times 7}$denotes a 7x7 convolution with a stride of 1, BN(·) denotes the bulk normalization layer, and  σ(·) stands for the activation function.

Building upon this, we have introduced a globally centralized rule for common feature pyramids in a top-down manner. This involves utilizing explicit visual center information acquired from the deepest layers to adjust the shallow features at the front end.In comparison with existing feature pyramids, EVCBlock not only demonstrates the capability to capture global long-term dependencies but is also efficient in obtaining comprehensive yet distinctive feature representations.

### YOLOv7 introduces the NAM attention mechanism

In real vehicle-pedestrian detection situations, existing networks are often unable to attend to the essential features of vehicle-pedestrians. For this reason, this paper uses an attention mechanism based on the YOLOv7 web to increase expressiveness, focus on crucial elements, and suppress unnecessary features. Self-attention-based modules have a significant boost on sequence learning tasks. Normalized Attention Module (NAM Attention), without the need for additional parameters, can reduce the weight of poor-performing features. This method employs sparse weight penalties for attention modules, leading to more efficient computation of these weights while maintaining the same performance.

The NAM attention mechanism works as follows

A module integration approach from CBAM was used, and the channel and spatial attention submodules were redesigned. Subsequently, at the end of each network block, it is necessary to embed a NAM module, while for residual networks, it should be incorporated at the end of the remnant structure. Within the channel attention sub-module, the scaling factor from Batch Normalization (BN) [41], as shown in equation (1), is employed. These scaling factors are used to measure the channel variance and reflect their significance.


$$B_{out}=BN\left(B_{in}\right)=\;\gamma \frac{B_{in}-\mu_{B}}{\sqrt{\sigma_{B}^{2}+\epsilon }}+\;\beta$$ (1)

Where $\;\mu_{B}\;$ is the mean, and $\;\sigma_{B}^{2}\;$ is the variance;  γ and  β are trainable affine transformation parameters (scale and shift).


$$M_{c}=sigmoid\left(W_{\gamma }\left(BN\left(F_{1}\right)\right)\right)$$ (2)

**Fig 3:**
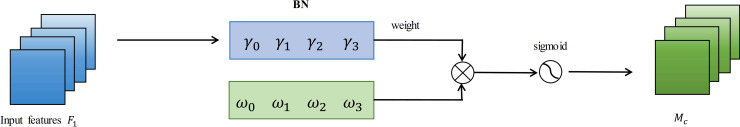
Channel attention mechanism.


As shown in (Fig 3) Equation (2), the attention module for the channel consists of output features $M_{c}$, a scaling factor γ for each channel and the weights are $W_{\gamma }=\gamma_{i}/\sum_{j=0}\gamma_{i}$. In addition, the importance of spatial features is evaluated using the scaling factor of BN, which is employed in the spatial dimension.

$$M_{s}=sigmoid\left(W_{\lambda }\left(BN_{s}\left(F_{2}\right)\right)\right)$$ (3)

$$Loss=\;\sum_{\left(x,y\right)}l\left(f(x,W),y\right)+p\sum g(\gamma )+p\sum g(\lambda )$$ (4)

**Fig 4:**
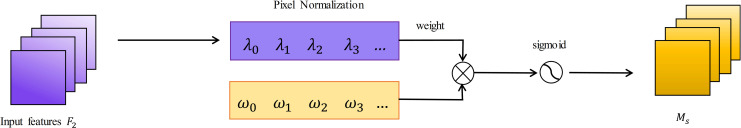
Spatial attention mechanism.

If the weights of spatial attention are obtained by using the identical method of normalization for each pixel in the space, as shown in equation (3), it is called pixel normalization. (Fig 4) illustrates the corresponding spatial attention sub-module, with output as $M_{s}$, the scale factor as λ, and the weight as $W_{\lambda }=\lambda_{i}/\sum_{j=0}\lambda_{j}$. To mitigate the impact of smaller weight values in the loss function, we have introduced a regularization term, which is specified in equation (4) [42].

In this paper, NAM Attention is applied to both the SPP module and the FPN module, which is represented by the red NAM module in (Fig 1) In the SPP module, the attention mechanism is utilized to adapt the weights of the feature maps of different scales to enhance the network’s attention to small target features, such as vehicles and pedestrians, so as to increase the detection capability. In the FPN module, the attention mechanism is used to adapt the weights of feature maps at various layers to improve detection precision and robustness.

### Optimizing backbone networks using deformable convolution networks (DCN-v2)

To improve the feature extraction capability of the backbone in a complex traffic environment, we optimized the backbone network by using DCN-v2 (deformable convolutional network). DCN-V2 is an improved convolutional operation that adds modulation modules and the use of multiple modulated DCN modules to DCN, which can be used in object detection tasks to enhance the accuracy of the detector. While conventional convolutional operations only consider fixed sampling positions, DCN-v2 (Deformable ConvV2) believes that the sampling position of each location on the feature map can be automatically adjusted based on spatial transformations on the feature map, thus capturing the shape and texture of the target more accurately.

DCN-v2 spatially transforms the input characteristic map to obtain the offset of each position, adjusts the sampling position of each class according to the balance, performs a convolution operation on the features obtained from the sampling, and weighted fusion of the convolution results to get the final feature representation. This makes the acceptance domain no longer restricted to a fixed extent but more flexible and adapted to variations in target geometry. DCN-v2 can adequately detect complex settings.

**Fig 5:**
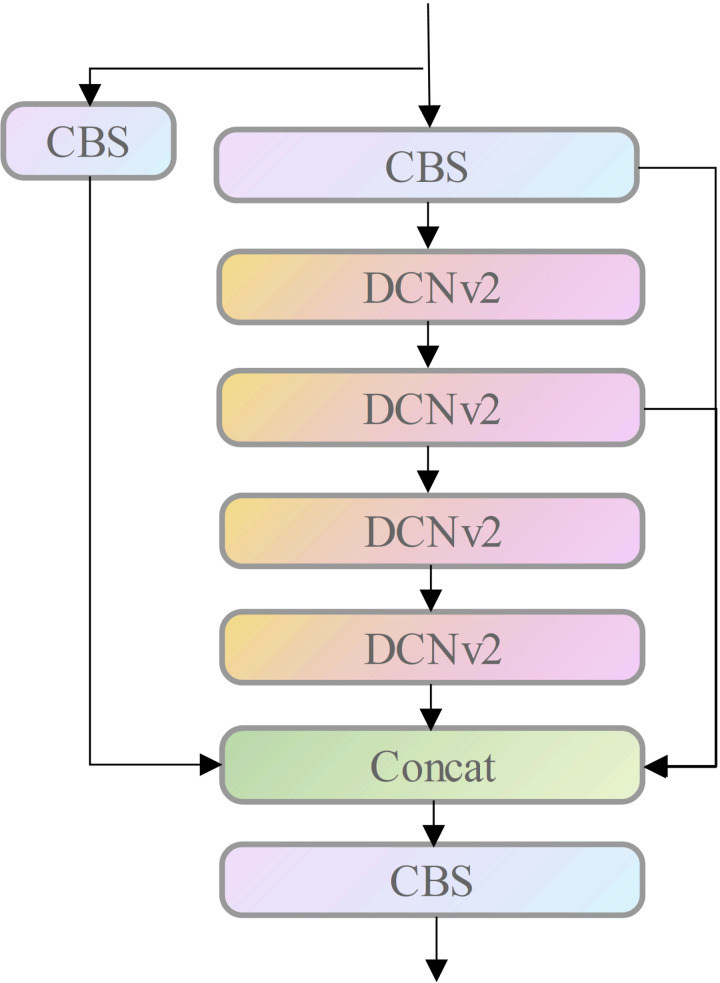
Adding attention mechanism positions.

In practical applications, issues such as the computational efficiency of multiple DCN-v2 layers and the stability of model training also need to be considered. Therefore, to balance effectiveness and efficiency, the article only replaces the 3 × 3 convolutional layer with DCN-v2 in the final stage. As shown in (Fig 5).

## Experiments

**Fig 6:**
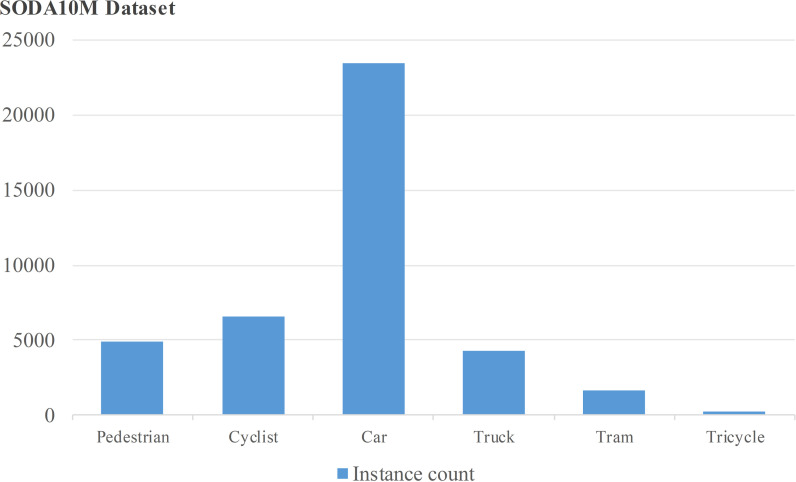
SODA10M dataset instance distribution.

Whether the target detection model is suitable for practical application scenarios mainly depends on whether its network structure is well-designed and has a certain degree of security; second, the dataset selected during training should be representative. To demonstrate our improved YOLOv7 network algorithm for target detection in complex traffic scenarios, we choose the high-quality (1080P+) driving scene SODA 10M dataset collected through the crowdsourcing distribution mode, which is a semi-supervised or self-supervised 2D benchmark dataset for evaluating target detection performance in complex scenes. This dataset will be able to cover, as comprehensively as possible, most of the fundamental road scene detection algorithms under the performance test for light changes, occlusion changes, scale changes, and other natural traffic scenes to shoot the corresponding images and videos. It mainly contains ten million images of unlabeled road scenes with rich diversity and 20,000 images with labels collected from thirty-two cities, while the photos have a variety of different road scenes (city, highway, urban/rural roads, parks), weather (sunny, cloudy, rainy, snowy), and periods (daytime, nighttime, dawn/dusk). Its abundant diversity ensures its generalization performance as a self-supervised pre-training dataset and also as semi-supervised additional data for subsequent autonomous driving tasks. The statistical characteristics of its data are shown in (Fig 6) [31].

**Fig 7:**
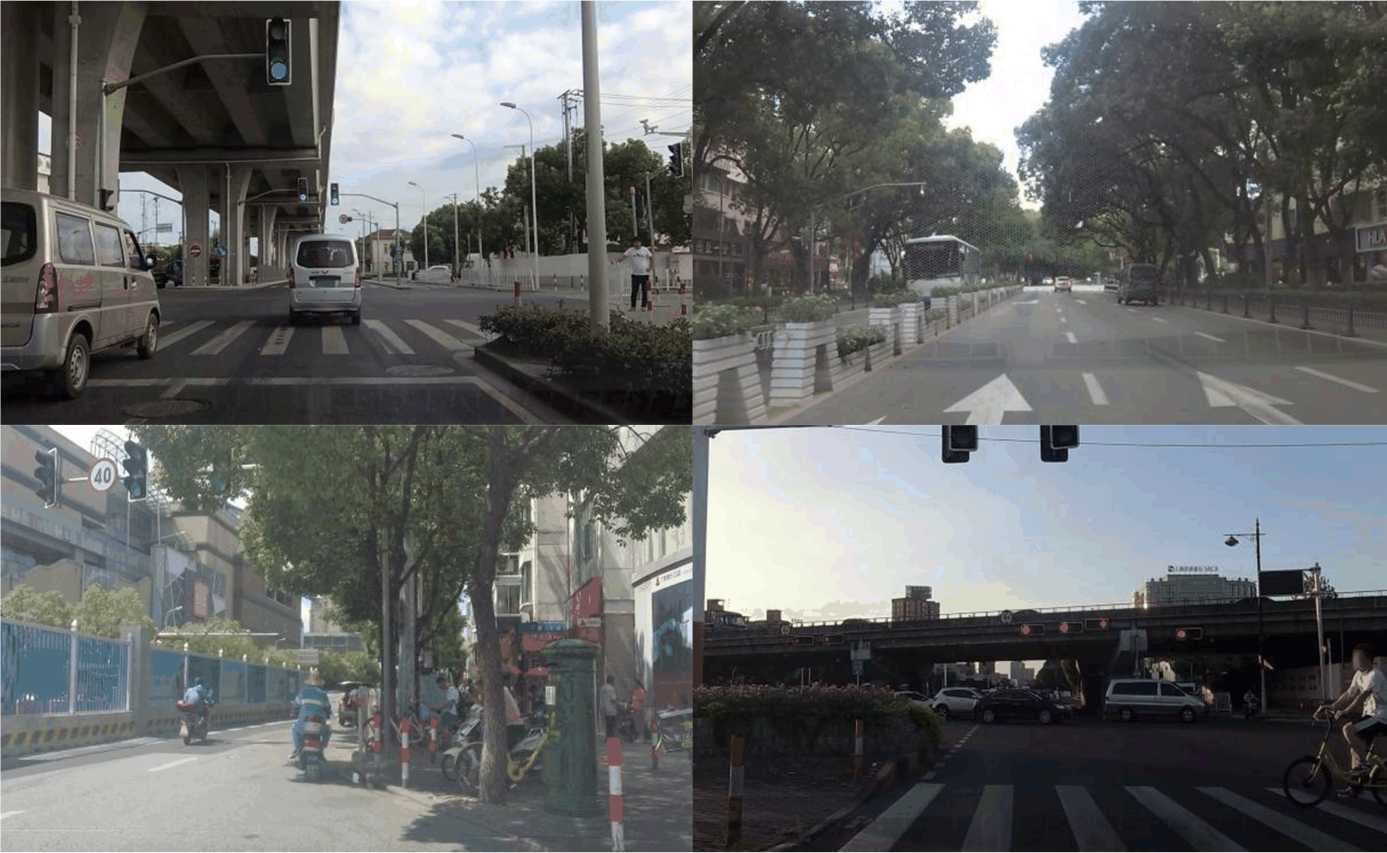
Example images of the SODA10M dataset.

Examples of images from the SODA10M dataset are illustrated in (Fig 7). The diverse range ensures its ability to generalize as a self-supervised pre-training dataset and also serves as semi-supervised supplementary data for subsequent autonomous driving tasks.

The SODA10M dataset includes six categories from 32 cities across the country, including six human-vehicle scenarios: pedestrians, bicycles, cars, trucks, trams, and tricycles. The entire dataset is partitioned into a training set, a validation set, and a test set with respective proportions of 7/10, 1/10, and 1/5.

The assessment involves a quantitative comparison between the dataset’s prediction and the real one, using mean average precision (mAP) as the metric for accuracy assessment, enabling a fair comparison with the original YOLOv7 network. To determine TP, FP, and FN for a specific target actual frame and generated prediction frame, an intersection (IoU) threshold of 0.5 is applied, following which precision and recall are calculated using equations (5) and (6), respectively.


$$Precision=\frac{TP}{TP+FP}$$ (5)

$$Recall=\frac{TP}{TP+FN}$$ (6)

After obtaining the precision and recall, the AP for each category is obtained according to the Pascal VOC 2012 calculation method, and then the ultimate indicator mAP is calculated. It is worth noting that the IoU threshold can be raised to 0.9 or 0.95 for comparison, highlighting the benefits of our high-precision localization.

During the experiment, we utilized the entire training and validation sets to train the enhanced detector, reserving the unlabeled test set for evaluation to maintain fairness in the experiment. Prior to the workout, certain essential preprocessing tasks are carried out. It is mainly described in Section 2 of this paper.

During the training phase, we employ YOLOv7.yaml as the backbone feature extraction network; the input image size is 640 × 640, the total training epoch is set to 150, and the batch size of each epoch is set to 8. The SGD optimizer’s initial learning rate is set at 0.01, with a weight decay of 0.001, and all other hyperparameters are set as defaults. To prevent overfitting during training, we primarily used two data enhancement methods: rotating horizontally and mosaics. All our experiments were conducted using an NVIDIA GeForce RTX 3090 GPU for both training and reasoning, featuring NMS in the post-processing stage. It is important to highlight that the ablation research solely compares the models’ accuracy on the test set.

### Mosaic data amplification and resolution

In order to confirm the efficacy of mosaic data augmentation for detecting small targets, we selected the Gaussian radius with the most favorable detection effect and employed two methods: one without mosaic and the other with mosaic data enhancement. The analysis results presented in Table 1 indicate that the utilization of mosaic data enhancement leads to a one to two percentage point increase in detection accuracy compared to not using mosaic data enhancement, albeit with a slight increase in training convergence time. It is a widely acknowledged fact that higher image resolution yields richer information for the model to learn from, resulting in improved target detection accuracy. In order to verify the impact of image size on the detection performance, we configured the training input size to 640 and 800. Additionally, we removed some images containing an excessive number of categories to maintain consistency across categories. The experimental findings are detailed in Table 1. The results indicate that the detection performance is superior when using a training set size of 800 compared to 640. Furthermore, setting the training set size to 1280 leads to improved detection results. However, it’s important to note that this size was not evaluated in this paper due to experimental equipment limitations.

**Table 1:** Mosaic and different size effects.

Techniques		Different skill sets	
**Input_size:640**	√	√	–
**Input_size:800**	–	–	√
**Mosaic**	–	√	√
**mAP(%)**	71.2	72.0	72.5

### Attentional mechanisms and the performance of convolution

**Fig 8:**
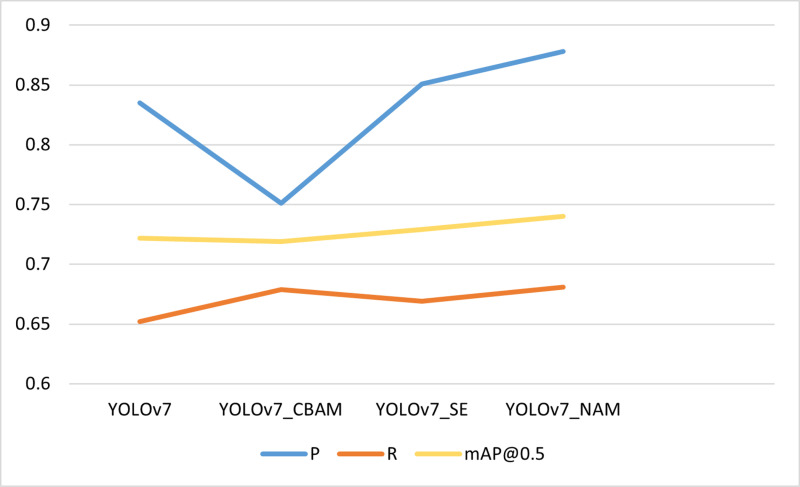
Shows a visualization of Table 2.

To validate our improvement of YOLOv7_ned’s attention mechanism, we conducted the appropriate experiments. First, we compared the effects of models that incorporated the NAM attentional mechanism with other attentional mechanisms and those that did not incorporate the attentional mechanism on SODA10M. The experimental results are presented in Table 2. From the table, it can be seen that the effect is not satisfactory after adding the CBAM attention mechanism; after adding the SE attention mechanism, the mAP value is increased by about 1 percentage point; after adding the NAM attention mechanism, the mAP is increased by about 2 percentage points; and it can be seen that the NAM attention mechanism is more effective in focusing on the important features of vehicle pedestrians. The Visualization of Table 2 is shown in the Fig 8.

**Table 2:** Attention module performance.

Method	ATT	P	R	mAP@0.5
YOLOv7	–	0.835	0.652	0.722
YOLOv7_CBAM	CBAM	0.751	0.679	0.719
YOLOv7_SE	SE	0.851	0.669	0.729
YOLOv7_NAM	NAM	0.878	0.681	0.740

**Fig 9:**
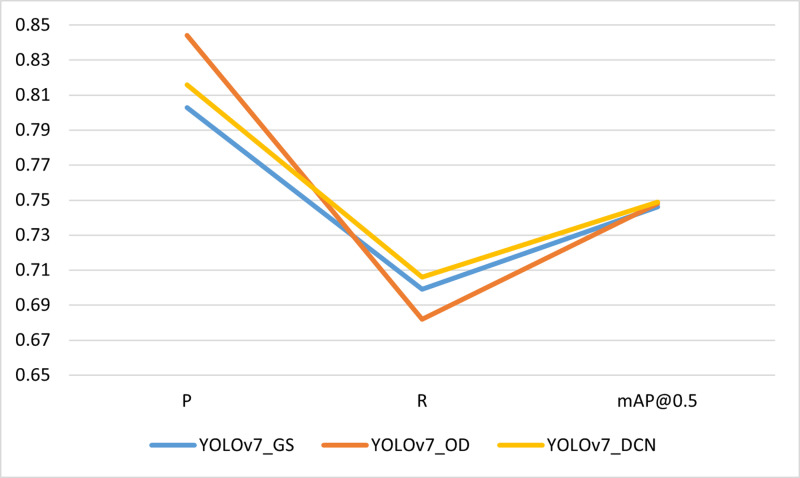
Shows a visualization of **Table 3**.

Then, we also compared the impact of using different convolutions on detection accuracy, as illustrated in Table 3. The Visualization of Table 3 is shown in the Fig 9.

**Table 3:** Convolutional detection results.

Method	Conv	P	R	mAP@0.5
YOLOv7_GS	GSConv	0.803	0.699	0.746
YOLOv7_OD	ODConv2d	0.844	0.682	0.748
YOLOv7_DCN	DCNv2	0.816	0.706	0.749

From the table, it can be noticed that when using DCNv2 (Deformable ConvV2), the mAP value is increased by about three percentage points, which significantly improves the target detection accuracy and works better compared to the other two convolutions.

Since we improved YOLOv7, we initially compared the performance of the two algorithms before and after the enhancement on the SODA10M dataset, as shown in Table 4.

**Table 4:** Performance comparison using the SODA10M test set.

SODA10M	Pedestrian	Cyclist	Car	Truck	Tram	Tricycle	mAP@0.5
YOLOv7(AP@0.5)	0.91	0.795	0.842	0.857	0.626	0.303	0.722
YOLOv7_ned(AP@0.5)	0.919	0.798	0.862	0.850	0.624	0.501	0.759

**Fig 10:**
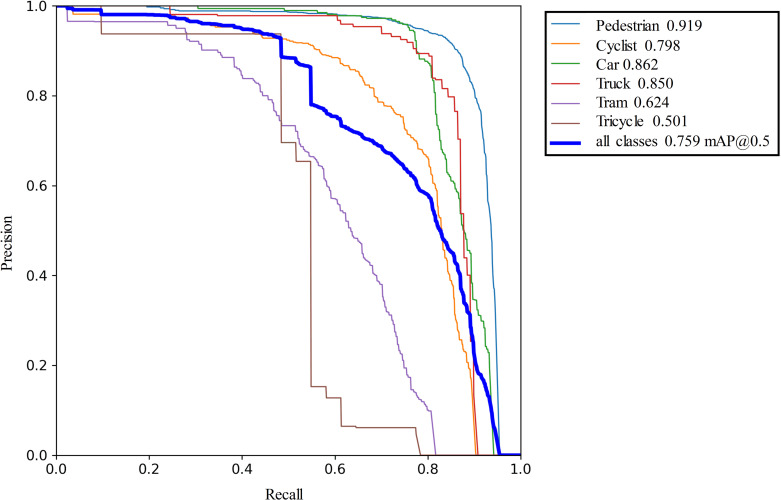
Precision-recall curves for the YOLOv7-ned model on the SODA10M dataset.

As shown in (Fig 10), in the SODA10M test set, the mAP value of YOLOv7 is 0.722, while that of the proposed YOLOv7-ned is 0.759, which is a 3.7% improvement in detection accuracy. By analyzing the changes in the average precision (AP) values for each class using YOLOv7 and YOLOv7-ned, our findings demonstrate that in complex traffic detection scenarios, YOLOv7-ned has significantly enhanced the detection accuracy of small objects. At the same time, YOLOv7-ned improved performance results by 2.4% and 1.1% when detecting objects such as cars and pedestrians, which vary in size (i.e., small in the distance and large up close). When dealing with objects characterized by large bounding boxes, such as trucks, streetcars, and non-motorized vehicles, YOLOv7-ned achieves slightly higher detection accuracy. From these results, we can reasonably conclude that the proposed YOLOv7-ned sufficiently enhances the detection accuracy of small objects without compromising the detection accuracy of larger, more common objects.

### Comparison of detection performance with other algorithms

**Fig 11:**
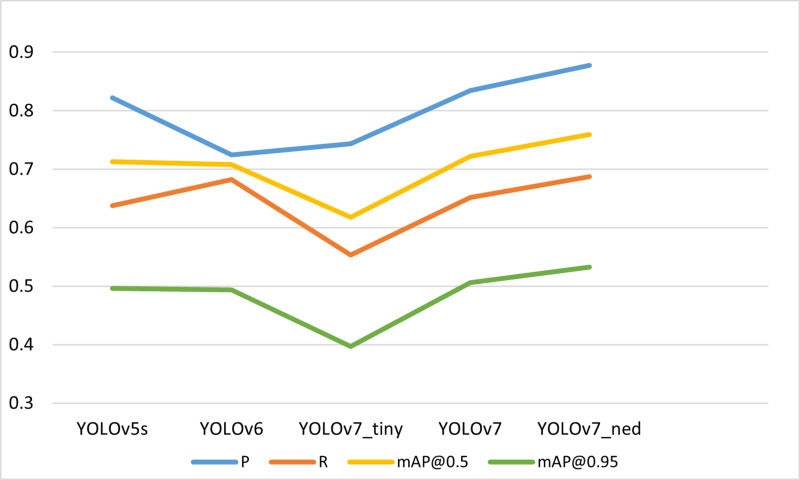
Shows a visualization of **Table 5**.

In our efforts to underscore the superiority of the proposed algorithms, we conducted a comparison with well-known target detection models (such as YOLOv7, YOLOv6, YOLOv5s, etc.), as detailed in Table 5. In the experiments with SODA10M test data, the obtained results are trained and evaluated using the officially released code of each algorithm. The Visualization of Table 5 is shown in the Fig 11.

**Table 5:** Performance comparison of target detection models on the SODA10M dataset.

Method	P	R	mAP@0.5	mAP@0.95	FPS
YOLOv5s	0.822	0.638	0.713	0.496	111
YOLOv6	0.725	0.682	0.708	0.494	94
YOLOv7_tiny	0.744	0.553	0.618	0.397	143
YOLOv7	0.835	0.652	0.722	0.506	102
YOLOv7_ned	0.878	0.687	0.759	0.533	110

As depicted in the table, the YOLOv7_ned model surpasses other detection algorithms, and its speed performance is also assessed by comparing its FPS metrics with those of other target detection models. It is important to note that while the detection speed of the smaller model will be further accelerated, its accuracy often fails to reach our desired standard for target recognition within complex traffic scenes, and the error will be further increased.

**Fig 12:**
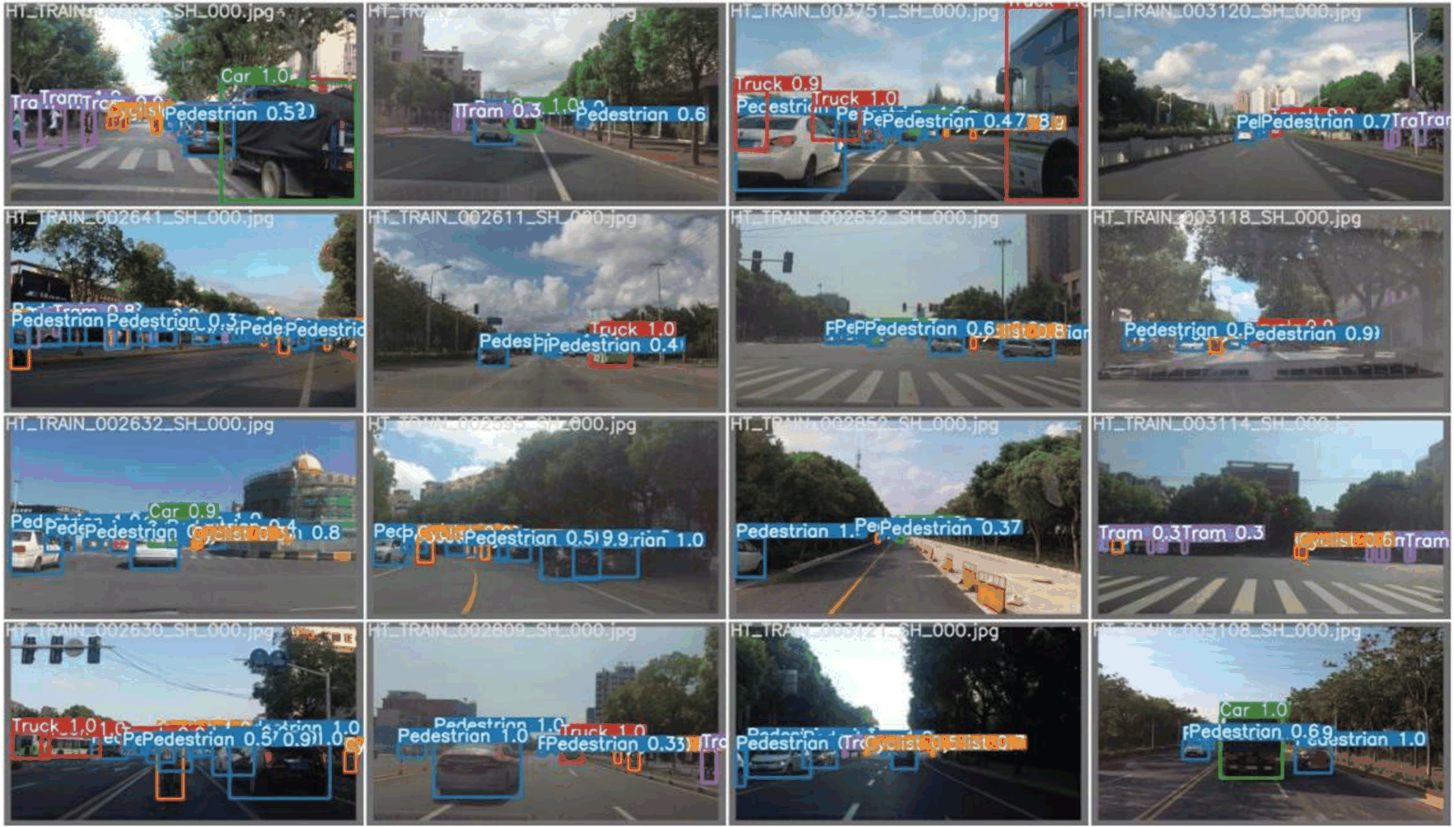
YOLOv7-ned detection results in complex traffic scenarios.

Therefore, our approach successfully applies the ideas of YOLOv7 to target detection techniques in complex traffic scenes. (Fig 12) shows the intuitive results.

## Discussion

In this paper, we present a vehicle and pedestrian detection algorithm that can be applied in embedded systems based on the YOLOv7 network using dimensional clustering analysis, multiscale training, and improvement of the feature extraction network. Although our approach is straightforward and barely increases the model’s parameter count, many tasks still need to be continued in depth and improved during the research process. For example, when processing real-time captured road scene videos on the mobile side, it is found that the detection model proposed in this paper tends to ignore the correlation information between the front and back frames of the video. Therefore, the introduction of a target tracking algorithm, which utilizes the inter-frame correlation information to enhance the real-time performance of target detection, is considered to track the detected targets in the video sequences for a period of time. In contrast to other approaches, our enhanced algorithm necessitates fewer hardware devices, achieves swifter computing speeds, and represents a more economical solution. Through the analysis of key parameters, we have also validated the optimal range of parameter combinations. This will assist others in replicating our results more efficiently and making significant enhancements.

## Conclusions

Aiming at the current vehicle-pedestrian detection algorithms with low accuracy in complex road scenes, many model parameters, and difficulty to deploy in embedded systems, we propose a convolutional neural network-based vehicle-pedestrian detection algorithm that not only meets the real-time and robustness requirements of detection in complex road scenarios but also improves the accuracy of vehicle-pedestrian detection. As an exploratory work on target detection in difficult traffic scenes, this paper has great reference value. Our study introduces the YOLOv7_ned algorithm, which is well-suited for addressing the challenges of complex traffic target detection in practical applications. In addition, this straightforward enhancement can be leveraged across various advanced detection algorithms to extend their applicability while maintaining a reasonable level of accuracy. Our model achieves 75.9% mAP on the SODA 10M dataset. Currently, the algorithm in this paper is unable to achieve the desired detection results for some unconventional types or structures of vehicles and pedestrians with particular postures that appear in natural road scenes. We analyze the reason for this, as the training samples of the model do not contain the vehicle and pedestrian targets that have the above features. In the time ahead, we may continue to optimize the training samples of the model so that it can have a more comprehensive vehicle and pedestrian detection range and improve the competitiveness of our algorithm.
